# Diaphragmatic hernia after pediatric liver transplantation: graft-specific incidence, clinical characteristics, and outcomes

**DOI:** 10.3389/fped.2026.1871728

**Published:** 2026-08-11

**Authors:** Cansu Altuntaş, Alaaddin Aydın, Mey Talip, Gülden Özek, Taylan Şahin, Ali Koçyiğit, Eryiğit Eren, Mehmet Tokaç, Ayhan Dinçkan

**Affiliations:** 1 Pediatric Gastroenterology, Medical Faculty, Istinye University, Istanbul, Türkiye; 2 General Surgery, Medical Faculty, Istinye University, Istanbul, Türkiye; 3 Pediatric Intensive Care Unit, Medical Faculty, Istinye University, Istanbul, Türkiye; 4 Radiology, Istinye University Bahcesehir Liv Hospital, Istanbul, Türkiye; 5 Anesthesiology, Medical Faculty, Istinye University, Istanbul, Türkiye; 6 Interventional Radiology, Istinye University Bahcesehir Liv Hospital, Istanbul, Türkiye

**Keywords:** liver transplantation, diaphragmatic hernia, child, left lateral segment graft, graft-to-recipient weight ratio, postoperative complications, living donors

## Abstract

**Background:**

Diaphragmatic hernia (DH) is a rare but clinically important complication following pediatric liver transplantation.

**Methods:**

This retrospective single-center study evaluated consecutive pediatric liver transplant episodes. Clinical characteristics, timing, management, and outcomes of DH were assessed. Transplant episodes with and without subsequent DH were compared. The cumulative incidence of DH was estimated using a competing-risk approach, with death before DH diagnosis treated as a competing event.

**Results:**

Diaphragmatic hernia occurred in 16 of 112 pediatric liver transplant episodes (14.3%). All cases occurred following left lateral segment (LLS) transplantation, corresponding to an incidence of 25.0% (16/64) among LLS transplant episodes, whereas no cases occurred after other graft types. Recipients who developed DH were younger at transplantation and had higher graft-to-recipient weight ratio (GRWR) values than those without DH. Approximately half of DH cases were diagnosed within the first 3 postoperative months, and most within the first year. In competing-risk analysis, the cumulative incidence of DH was 7.2% at 3 months, 10.0% at 6 months, and 11.9% at 12 months. Respiratory distress was the most common presentation. All patients underwent surgical repair, and dual mesh reinforcement was used in most cases. Severe postoperative complications were frequent, recurrence occurred in two patients, and no deaths were directly attributable to DH.

**Conclusion:**

Diaphragmatic hernia is an unexpected and serious complication following pediatric liver transplantation. In our cohort, DH occurred exclusively after LLS transplantation, and affected recipients were younger and had higher GRWR values than those without DH; however, these characteristics are closely interrelated and their independent contributions could not be determined. Most cases were diagnosed early after transplantation, emphasizing the importance of clinical vigilance, timely diagnosis, and prompt surgical management.

## Introduction

Diaphragmatic hernia (DH) is a rare but potentially life-threatening complication following liver transplantation, particularly in pediatric recipients (1, 2). Clinical presentation varies widely, ranging from incidental, asymptomatic findings to acute emergencies characterized by severe abdominal pain, vomiting, or respiratory distress. In addition, recurrence of DH after successful surgical repair has been reported in the literature (3).

Several risk factors have been proposed for the development of DH after liver transplantation, including malnutrition, loss of the liver's anatomical barrier function between the diaphragm and the abdominal cavity, increased intra-abdominal pressure, and diaphragmatic injury during transplantation (2, 4).

Prompt recognition of DH, especially in acute clinical settings, is crucial to prevent serious complications and mortality (5). However, as DH may also present in a delayed or asymptomatic manner, increased clinical awareness and consideration of routine screening strategies are warranted (6).

In this study, we aimed to investigate the clinical characteristics, timing of presentation, and postoperative outcomes of pediatric liver transplant recipients diagnosed with DH. By analyzing these cases, we sought to identify high-risk groups and to emphasize the importance of early recognition and preventive strategies for this rare but serious complication.

## Materials and methods

### Study design and patient population

This retrospective observational study was conducted at the Organ Transplantation Unit of Istinye University, Istanbul. All consecutive pediatric liver transplant episodes performed between January 2018 and March 2025 were included. Retransplantation procedures were analyzed as separate transplant episodes because postoperative DH risk was considered specific to each surgical exposure and graft.

### Data collection

All cases of DH diagnosed either symptomatically or incidentally during follow-up were included in the study group. For all transplant recipients, collected data included age and weight at the time of transplantation, weight standard deviation score (SDS), primary liver disease etiology, type of transplanted liver lobe, and graft-to-recipient weight ratio (GRWR). Post-transplant gastrointestinal complications were also documented.

For patients diagnosed with DH (DH group), additional variables were reviewed, including the timing of hernia onset, presenting symptoms, herniated intra-abdominal organs, details of surgical repair (including mesh use), recurrence, and postoperative outcomes classified according to the Clavien–Dindo classification.

### Diagnosis and ethical approval

All data were collected retrospectively from medical records and surgical reports. The diagnosis of DH was confirmed by radiological imaging and/or intraoperative findings. The study was approved by the Istinye University Human Research Ethics Committee (file number 25–43, April 22, 2025).

### Statistical analysis

All statistical analyses were performed using IBM SPSS Statistics version 25.0 (IBM Corp., Armonk, NY, USA). The Shapiro–Wilk test was used to assess the normality of continuous variables, and Levene's test was used to evaluate homogeneity of variances. Categorical variables were summarized as frequencies and percentages, whereas continuous variables were reported as mean ± standard deviation or median with interquartile range, as appropriate according to data distribution.

Between-group comparisons of continuous variables were performed using the independent-samples Student's t-test when parametric assumptions were met; otherwise, the Mann–Whitney U test was used. Categorical variables were compared using Fisher's exact test. The cumulative incidence of DH after transplantation was estimated within a competing-risk framework, with death before DH diagnosis treated as a competing event. Transplant episodes without DH or death were administratively censored on June 30, 2025. In the case of retransplantation, the preceding transplant episode was censored at the date of subsequent transplantation. Cumulative incidence estimates were reported at 3, 6, and 12 months after transplantation.

All tests were two-sided, and a *p* value < 0.05 was considered statistically significant.

## Results

### Patient characteristics

Between January 2018 and March 2025, a total of 112 pediatric liver transplant episodes were evaluated. All but three patients received variant grafts. The graft distribution was as follows: left lateral segment in 64 (57.1%), left lobe in 30 (26.8%), right lobe in 14 (12.5%), whole liver in 3 (2.7%), and right posterior segment in 1 (0.9%). The most common indication for transplantation was biliary atresia, followed by progressive familial intrahepatic cholestasis (PFIC). The mean age at transplantation was 5.2 ± 4.7 years, the mean body weight was 19.4 ± 13.1 kg, and the mean weight standard deviation score (SDS) was −1.3 ± 1.4. The median follow-up duration for the entire cohort was 26.3 months (IQR, 8.9–45.6 months).

### Incidence and clinical characteristics of diaphragmatic hernia

Among the entire cohort, 16 patients (14.3%) developed diaphragmatic hernia (DH). DH occurred exclusively among LLS recipients, corresponding to an incidence of 25.0% (16/64), with no cases among other graft types. The mean age at transplantation in the DH group was 1.27 ± 1.42 years, with an equal sex distribution (50% female and 50% male). Biliary atresia was the predominant underlying diagnosis (68.8%), and three patients (18.8%) had undergone Kasai portoenterostomy before transplantation. The median follow-up duration was 28.8 months (IQR, 9.2–42.5). Four patients (25%) died during follow-up; however, none of the deaths were attributable to DH.

**Figure 1:**
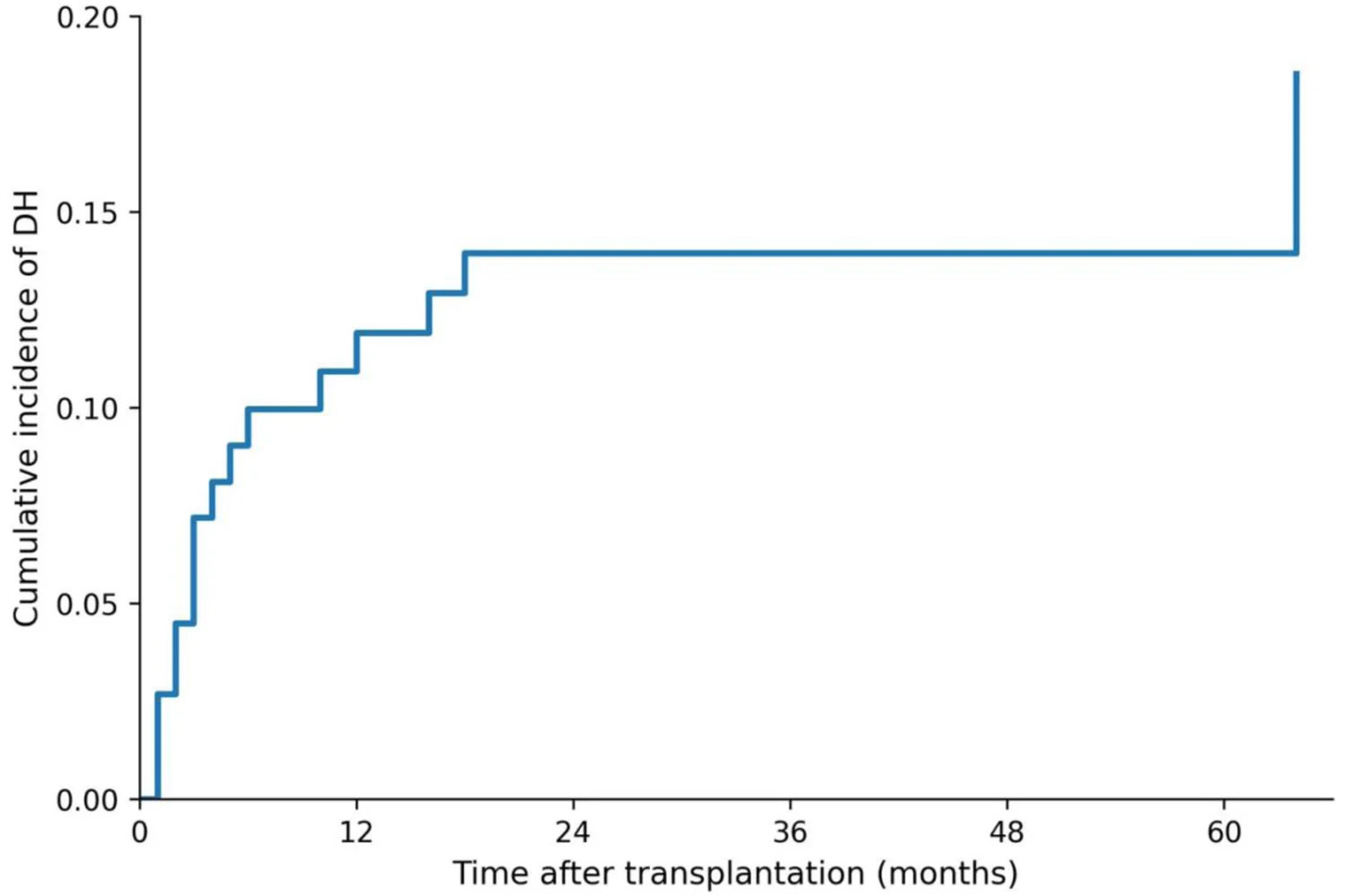
Cumulative incidence of diaphragmatic hernia after pediatric liver transplantation. Death before DH diagnosis was treated as a competing event.

Detailed individual patient data are provided in Supplementary Materials S1 and S2, while the baseline characteristics of the DH cohort are summarized in Table 1. In the DH group, the mean body weight at transplantation was 7.9 ± 2.07 kg, the mean weight SDS was −1.70 ± 1.26, and the mean graft-to-recipient weight ratio (GRWR) was 3.32 ± 0.57. Post-transplant gastrointestinal complications occurred in 11 patients (68.8%), most commonly gastrointestinal perforation (37.5%). Six patients (37.5%) required relaparotomy before the diagnosis of DH. The median interval between transplantation and DH diagnosis was 3.5 months (IQR, 2.0–14.0 months; range, 1–64 months), with approximately half of the DH cases diagnosed within the first 3 months after transplantation. In the competing-risk analysis, with death before DH diagnosis treated as a competing event, the cumulative incidence of DH was estimated at 7.2% at 3 months, 10.0% at 6 months, and 11.9% at 12 months (Figure 1).

**Table 1:** Demographic, clinical, and transplant-related characteristics of pediatric patients diagnosed with diaphragmatic hernia (*n* = 16).

Variable	*n* = 16
Transplant age (years), [mean ± SD]	1.27 ± 1.42
Range of Tx age (years), min – max	0.33–5.01
Gender	
Female	8 (50.0%)
Male	8 (50.0%)
Etiology	
Biliary atresia	11 (68.8%)
Acute hepatitis	1 (6.3%)
PFIC	2 (12.5%)
MSUD	1 (6.3%)
Tyrosinemia	1 (6.3%)
Follow-up time (months), [median (IQR)]	28.8 (9.2–42.5)
Mortality	4 (25.0%)
KASAI	3 (18.8%)
Graft weight (g), [mean ± SD]	258.1 ± 60.9
Body weight during Tx (kg), [mean ± SD]	7.9 ± 2.07
Weight SDS, [mean ± SD]	−1.70 ± 1.26
GRWR, [mean ± SD]	3.32 ± 0.57
GIS complication	
No	5 (31.3%)
Perforation	6 (37.5%)
Bleeding	2 (12.5%)
Ileus-gastroparesis	3 (18.8%)
Post-tx repeated surgical intervention	6 (37.5%)
Diaphragmatic hernia diagnosis time (months), [median (IQR)]	3.5 (2.0–14.0)
Herniated segment	
Small intestine	6 (37.5%)
Colon and small intestine	8 (50.0%)
Roux-en-Y limb	2 (12.5%)
Symptoms in diagnosis	
Coincidental	2 (12.5%)
Respiratory distress	9 (56.3%)
Vomiting, restlessness	4 (25.0%)
Fever, infection	1 (6.3%)
Mesh usage	11 (68.8%)
Clavien-Dindo	
III	7 (43.7%)
IV	9 (56.3%)
Recurrence	2 (12.5%)

Data are presented as mean ± standard deviation or median (interquartile range), as appropriate. Tx, transplantation; SDS, standard deviation score; GRWR, graft-to-recipient weight ratio; PFIC, progressive familial intrahepatic cholestasis; MSUD, maple syrup urine disease; GIS, gastrointestinal; Post-tx, post-transplant.

### Clinical presentation and surgical management

The most frequently herniated contents were the colon and small bowel in combination (50.0%), followed by isolated small bowel (37.5%) and the Roux-en-Y limb (12.5%). Respiratory distress was the most common presenting symptom (56.3%), although gastrointestinal symptoms and incidental radiologic findings were also observed.

Following diagnosis, patients underwent surgical repair as soon as clinically feasible after hemodynamic stabilization. All procedures were performed through an abdominal approach. Dual mesh reinforcement was used in 11 patients (68.8%), while primary repair was performed in the remaining 5 patients (31.2%). Hernia recurrence occurred in 2 patients, both of whom had undergone mesh repair. According to the Clavien–Dindo classification, postoperative complications were grade IV in 9 patients (56.3%) and grade III in 7 patients (43.7%).

### Comparative analyses

Comparative analyses between transplant episodes associated with DH and those without DH are summarized in Table 2. Transplant episodes associated with DH involved significantly younger recipients at transplantation [median 0.86 years (IQR, 0.65–0.98) vs. 4.00 years (IQR, 1.08–10.04), *p* < 0.001], lower body weight [7.48 kg (IQR, 6.93–7.98) vs. 13.00 kg (IQR, 7.00–26.85), *p* = 0.006], and higher GRWR values [3.42% (IQR, 2.91–3.65) vs. 2.07% (IQR, 1.38–3.14), *p* < 0.001]. Mean weight SDS did not differ significantly between the groups (−1.70 ± 1.26 vs. −1.75 ± 1.89, *p* = 0.917).

**Table 2:** Comparison of demographic and transplant-related characteristics between transplant episodes associated with diaphragmatic hernia and those without diaphragmatic hernia.

Variable	DH group (*n* = 16)	Non-DH group (*n* = 96)	*p*-value
Age at transplantation (years), [median (IQR)]	0.86 (0.65–0.98)	4.00 (1.08–10.04)	**<0**.**001**^a^
Body weight at transplantation (kg), [median (IQR)]	7.48 (6.93–7.98)	13.00 (7.00–26.85)	**0**.**006**^a^
Weight SDS, [mean ± SD]	−1.70 ± 1.26	−1.75 ± 1.89	0.917^b^
GRWR, [median (IQR)]	3.42 (2.91–3.65)	2.07 (1.38–3.14)	**<0**.**001**^a^
Follow-up duration (months), [median (IQR)]	28.8 (9.2–42.5)	26.3 (8.9–46.5)	**–**

a Mann Whitney U test, ^b^Student's t test. A two-sided *p* value < 0.05 was considered statistically significant. SDS, standard deviation score; GRWR, graft-to-recipient weight ratio.

Bold values indicate statistically significant differences between groups (*p* < 0.005).

Because all DH cases occurred after LLS transplantation, a subgroup analysis restricted to LLS transplant episodes was performed to determine whether the observed associations persisted within this graft category (Table 3). Compared with LLS transplant episodes without subsequent DH, those associated with DH involved significantly younger recipients [median 0.86 years (IQR, 0.65–0.98) vs. 2.64 years (IQR, 1.05–6.06), *p* = 0.002], lower body weight [7.48 kg (IQR, 6.93–7.98) vs. 11.00 kg (IQR, 6.80–18.13), *p* = 0.026], and higher GRWR values [3.42% (IQR, 2.91–3.65) vs. 2.24% (IQR, 1.37–3.29), *p* = 0.002]. Mean weight SDS was comparable between the groups (−1.70 ± 1.26 vs. −1.66 ± 1.68, *p* = 0.931).

**Table 3:** Comparison of demographic and transplant-related characteristics between left lateral segment graft recipients with and without diaphragmatic hernia.

Variable	LLS recipients with DH (*n* = 16)	LLS recipients without DH (*n* = 48)	*p*-value
Age at transplantation (years), [median (IQR)]	0.86 (0.65–0.98)	2.64 (1.05–6.06)	**0**.**002**^a^
Body weight at transplantation (kg), [median (IQR)]	7.48 (6.93–7.98)	11.00 (6.80–18.13)	**0**.**026**^a^
Weight SDS, [mean ± SD]	−1.70 ± 1.26	−1.66 ± 1.68	0.931^b^
GRWR, [median (IQR)]	3.42 (2.91–3.65)	2.24 (1.37–3.29)	**0**.**002**^a^

a Mann Whitney U test, ^b^Student's t test. A two-sided *p* value < 0.05 was considered statistically significant. SDS, standard deviation score; GRWR, graft-to-recipient weight ratio.

Bold values indicate statistically significant differences between groups (*p* < 0.005).

## Discussion

Diaphragmatic hernia following pediatric liver transplantation is an uncommon but clinically significant complication, with reported incidences generally ranging between 0.74% and 3.3% in the literature (1–3, 6, 7). The overall incidence in our cohort was 14.3%, substantially higher than previously reported. However, this figure should be interpreted in the context of our center-specific case mix rather than as the expected incidence after pediatric liver transplantation in general. Because of limited deceased donor availability, more than 90% of transplantations at our institution are living donor procedures, and our center receives a high proportion of infants and small children requiring transplantation. Consequently, LLS grafts are frequently used. Importantly, all DH cases occurred after LLS transplantation, corresponding to an incidence of 25.0% (16/64) within this subgroup, whereas no cases occurred after other graft types. Thus, graft-stratified incidence provides a more clinically informative context for interpreting the high overall incidence observed in our cohort.

All DH cases were right-sided, consistent with previous reports (3, 6–11). Implantation of a left-sided graft removes the native liver's protective support of the right hemidiaphragm and creates a potential space that may facilitate visceral migration (3, 10–12). Thermal or mechanical injury during recipient hepatectomy has also been proposed as a contributor to diaphragmatic weakening (11, 13, 14). At our center, the Aquamantys™ bipolar sealer (BIS) has routinely been used during recipient hepatectomy. Of relevance, Kim et al. reported an increased incidence of diaphragmatic hernia associated with BIS use in living liver donors (14), raising the possibility that thermal injury related to energy-device use may contribute to diaphragmatic weakening. However, BIS exposure was not evaluated as a comparative risk factor in our cohort; therefore, no causal or independent association can be inferred from our data.

Patients who developed DH were younger and had higher GRWR values than those who did not. In the analysis restricted to LLS recipients, GRWR remained significantly higher among those who developed DH, indicating that the observed difference persisted within this graft category. Nevertheless, recipient age, body size, and GRWR are closely interrelated, and the limited number of DH events precluded multivariable analysis; their independent contributions therefore cannot be distinguished. Biologically, younger recipients may be more susceptible because of reduced diaphragmatic thickness and tissue fragility, whereas relatively large grafts may increase intra-abdominal pressure and diaphragmatic tension (1–3, 11). Additional intra-abdominal events may further contribute to DH development. Gastrointestinal complications before DH diagnosis occurred in 11 of 16 patients, and 6 underwent relaparotomy, suggesting that repeated intra-abdominal insults may increase diaphragmatic injury (1, 11). In contrast, prior Kasai portoenterostomy was present in only 3 patients and did not appear to act as a significant predisposing factor in our cohort.

Our observations suggest a possible “two-hit” mechanism for DH development. The first hit consists of chronic diaphragmatic weakening caused by loss of hepatic support following LLS transplantation together with possible intraoperative thermal or mechanical injury. The second hit is an acute increase in intra-abdominal pressure that precipitates herniation through the weakened diaphragm. In our cohort, six patients developed symptoms immediately following transient events known to increase intra-abdominal pressure, including coughing, crying during venipuncture, diarrheal illness, and air travel. Although these observations are descriptive, they provide clinically plausible support for this proposed mechanism.

Although malnutrition-related sarcopenia may theoretically contribute to diaphragmatic weakness (11, 15), no significant difference was observed in weight-for-age SDS between groups. This finding may reflect the limitations of weight-based indices in liver transplant patients, where factors such as organomegaly and ascites may obscure the true nutritional and muscle status (3). These observations suggest that sarcopenia and malnutrition alone are insufficient to explain DH development. Future studies using ultrasound-based diaphragm thickness or cross-sectional muscle measurements may better clarify this relationship (2, 10).

**Figure 2:**
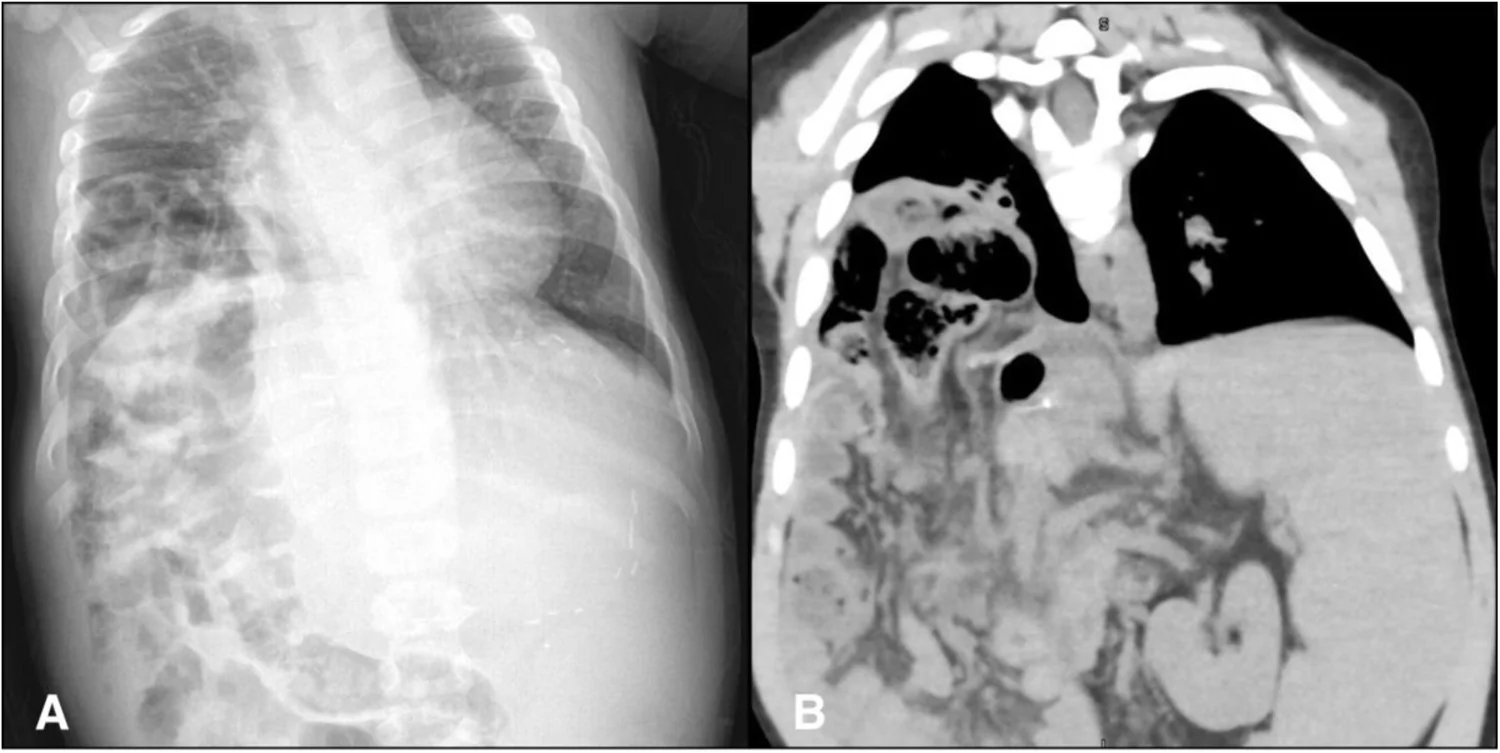
**(A)** chest radiograph demonstrating bowel gas within the right hemithorax. **(B)** Coronal CT image confirming herniation of abdominal contents through a diaphragmatic defect.

The temporal distribution of DH in our cohort suggests that the early postoperative period is particularly relevant for detection, with approximately half of cases diagnosed within the first 3 months and most within the first year. This early presentation is consistent with previous reports (8–10). However, a late asymptomatic case indicates that longer-term vigilance remains warranted. Respiratory distress was the most common presentation, but gastrointestinal and nonspecific symptoms, including vomiting and irritability, were also observed (2, 7, 11, 13, 16). Given that all cases occurred after LLS transplantation, surveillance may be particularly relevant in this subgroup. Clinical assessment may reasonably be prioritized during the first 3 postoperative months, with continued vigilance throughout the first year and thereafter. Rather than increasing routine chest radiography, diaphragmatic assessment could be incorporated into abdominal ultrasonography already performed during post-transplant follow-up, particularly by experienced pediatric radiologists (13). When clinical suspicion arises, chest radiography provides a simple initial imaging modality, whereas computed tomography may better define hernia contents and extent for preoperative planning (Figure 2). Greater awareness of the ultrasonographic appearance of acquired DH may facilitate earlier diagnosis while limiting cumulative radiation exposure.

From a management perspective, DH should be regarded as a surgical condition requiring prompt intervention upon diagnosis, as delay may result in severe complications such as bowel incarceration, obstruction, or perforation (8, 10, 11). In our center, patients underwent surgical repair as soon as clinically feasible after hemodynamic stabilization. All procedures were performed through an abdominal approach, allowing evaluation of associated gastrointestinal obstruction or adhesions (6). Dual mesh reinforcement was preferred over primary repair to potentially reduce the risk of recurrence. Although the optimal surgical technique remains debated, some studies suggest that recurrence rates may be higher following primary repair alone, supporting the use of mesh when feasible (13, 17). However, recurrence in two patients despite dual mesh repair in our cohort indicates that dual mesh use alone may not be sufficient to prevent recurrence, and further studies are needed to establish optimal surgical strategies.

Although no deaths were directly attributable to DH, morbidity following DH repair was substantial, with frequent severe postoperative complications, including Clavien–Dindo grade IV events. Similar findings have been reported in the literature, where DH has been associated with considerable morbidity and mortality, particularly in cases of delayed diagnosis (1, 5, 9, 10, 13, 18). These observations emphasize the need for management in experienced centers with appropriate surgical and intensive care expertise.

This study has several limitations. Its retrospective, single-center design limits external validity, and the high proportion of living donor LLS transplantations reflects our referral practice rather than the broader pediatric transplant population. Only 16 DH events occurred, precluding adequately powered multivariable analyses and limiting our ability to distinguish the independent effects of recipient age, body size, graft type, and GRWR. Surveillance was not protocolized throughout the study period, and imaging was primarily performed according to clinical indications and routine post-transplant follow-up; therefore, asymptomatic DH cases may have been missed, introducing potential detection and misclassification bias. Differences in follow-up duration may also have influenced ascertainment of this time-dependent outcome. Finally, objective measures of sarcopenia and diaphragmatic muscle quality were unavailable, preventing direct assessment of their potential contribution to DH development.

## Conclusion

Diaphragmatic hernia is an unexpected and serious complication following pediatric liver transplantation, with substantial morbidity. In our cohort, DH occurred exclusively after LLS transplantation, and affected recipients were younger and had higher GRWR values than those without DH, although these factors are closely interrelated. Most cases were diagnosed early after transplantation. Given the concentration of cases among LLS recipients, our findings support further evaluation of whether diaphragmatic assessment should be incorporated into routine postoperative follow-up, particularly during the early postoperative period. Larger multicenter studies are needed to clarify independent risk factors and define evidence-based surveillance strategies.

## Data Availability

The original contributions presented in the study are included in the article/Supplementary Material, further inquiries can be directed to the corresponding author.
