# Supplementary material for: Diaphragmatic hernia after pediatric liver transplantation: graft-specific incidence, clinical characteristics, and outcomes
Source: Front Pediatr. 2026 Aug 11;14:1871728. doi: 10.3389/fped.2026.1871728 (PMC13503309; doi:10.3389/fped.2026.1871728)
Supplement: Supplementary file 1 [file Table1.docx]

**Supplementary material 1.** Baseline demographic and transplant-related characteristics of pediatric patients diagnosed with diaphragmatic hernia.

| **Patient no** | **Transplant**  **age (years)** | **Gender** | **Etiology** | **Follow up**  **time (months)** | **Mortality** | **Prior KASAI** | **Graft type** | **Graft weight (g)** | **Body weight**  **during Tx (kg)** | **Weight SDS** | **GRWR** |
| --- | --- | --- | --- | --- | --- | --- | --- | --- | --- | --- | --- |
| 1 | 0.33 | F | Biliary atresia | 68 | No | No | Left lateral | 220 | 6.1 | -1.54 | 3.61 |
| 2 | 0.82 | M | Biliary atresia | 48 | No | No | Left lateral | 250 | 8.5 | -0.64 | 2.94 |
| 3 | 0.89 | M | Biliary atresia | 47 | No | No | Left lateral | 200 | 7.7 | -1.75 | 2.60 |
| 4 | 0.93 | F | Biliary atresia | 45 | No | Yes | Left lateral | 220 | 7.0 | -1.86 | 3.14 |
| 5 | 1.07 | F | Tyrosinemia | 26 | Yes | No | Left lateral | 275 | 7.5 | -1.98 | 3.67 |
| 6 | 0.93 | F | Biliary atresia | 4 | Yes | No | Left lateral | 220 | 7.8 | -0.95 | 2.82 |
| 7 | 1.02 | F | Biliary atresia | 42 | No | Yes | Left lateral | 195 | 5.6 | -4.07 | 3.48 |
| 8 | 0.69 | M | MSUD | 41 | No | No | Left lateral | 270 | 7.4 | -1.46 | 3.65 |
| 9 | 0.51 | M | Biliary atresia | 38 | No | No | Left lateral | 230 | 6.2 | -2.30 | 3.71 |
| 10 | 0.75 | F | PFIC | 11 | Yes | No | Left lateral | 240 | 7.3 | -1.00 | 3.29 |
| 11 | 4.74 | F | Biliary atresia | 34 | No | No | Left lateral | 400 | 10.0 | -4.32 | 4.00 |
| 12 | 0.97 | M | Acute hepatitis | 2 | Yes | No | Left lateral | 180 | 7.5 | -2.20 | 2.40 |
| 13 | 5.01 | M | PFIC | 22 | No | No | Left lateral | 360 | 14.5 | -1.95 | 2.48 |
| 14 | 0.36 | M | Biliary atresia | 18 | No | No | Left lateral | 250 | 7.4 | 0.34 | 3.36 |
| 15 | 0.83 | F | Biliary atresia | 13 | No | Yes | Left lateral | 320 | 9.0 | 0.36 | 3.56 |
| 16 | 0.50 | M | Biliary atresia | 10 | No | No | Left lateral | 300 | 6.7 | -1.85 | 4.48 |

Tx, transplantation; SDS, standard deviation score; GRWR, graft-to-recipient weight ratio; PFIC, progressive familial intrahepatic cholestasis; MSUD, maple syrup urine disease.
