# Supplementary material for: Diaphragmatic hernia after pediatric liver transplantation: graft-specific incidence, clinical characteristics, and outcomes
Source: Front Pediatr. 2026 Aug 11;14:1871728. doi: 10.3389/fped.2026.1871728 (PMC13503309; doi:10.3389/fped.2026.1871728)
Supplement: Supplementary file 2 [file Table2.docx]

**Supplementary material 2.** Post-transplant clinical course, presentation, and surgical outcomes of patients with diaphragmatic hernia.

| **Patient no** | **GIS**  **complication** | **Post-tx**  **repeated**  **surgical**  **intervention** | **Time of diagnosis**  **(months)** | **Herniated**  **segment** | **Symptoms**  **in diagnosis** | **Mesh Usage** | **Clavien**  **Dindo**  **classification** | **Recurrence** |
| --- | --- | --- | --- | --- | --- | --- | --- | --- |
| 1 | No | No | 64 | Colon +  Small intestine | Coincidental | Yes | IV | No |
| 2 | No | No | 4 | Small intestine | Respiratory distress | Yes | III | No |
| 3 | Perforation | Yes | 12 | Small intestine | Respiratory distress | No | III | No |
| 4 | Perforation | Yes | 3 | Colon +  Small intestine | Respiratory distress | Yes | IV | Yes |
| 5 | Ileus-gastroparesis | No | 6 | Colon +  Small intestine | Respiratory distress | Yes | IV | No |
| 6 | No | No | 1 | Small intestine | Respiratory distress | Yes | IV | No |
| 7 | Perforation | Yes | 18 | Colon +  Small intestine | Vomiting, restlessness | Yes | III | No |
| 8 | Perforation | Yes | 3 | Colon +  Small intestine | Vomiting, restlessness | Yes | III | No |
| 9 | No | No | 3 | Colon +  Small intestine | Respiratory distress | Yes | IV | No |
| 10 | Perforation | No | 1 | Small intestine | Respiratory distress | No | IV | No |
| 11 | No | No | 16 | Roux-en-Y limb | Vomiting, restlessness | Yes | III | No |
| 12 | Perforation | Yes | 1 | Small intestine | Respiratory distress | No | IV | No |
| 13 | Ileus-gastroparesis | No | 10 | Colon +  Small intestine | Vomiting, restlessness | Yes | III | No |
| 14 | Bleeding | Yes | 2 | Roux-en-Y limb | Respiratory distress | No | IV | No |
| 15 | Bleeding | No | 5 | Small intestine | Fever, infection | No | III | No |
| 16 | Ileus-gastroparesis | No | 2 | Colon +  Small intestine | Coincidental | Yes | IV | Yes |

GIS, gastrointestinal; Post-tx, post-transplant; Clavien–Dindo classification refers to postoperative complication grading.
